# Retroperitoneal lymph node dissection for testicular cancer is a demanding procedure: detailed real-life data of complications and additional surgical procedures in 295 cases

**DOI:** 10.1007/s00345-023-04516-7

**Published:** 2023-07-25

**Authors:** Stefanie Latarius, Steffen Leike, Holger Erb, Juliane Putz, Angelika Borkowetz, Christian Thomas, Martin Baunacke

**Affiliations:** Department of Urology, Medical Faculty Carl Gustav Carus, TU Dresden, Fetscherstr. 74, 01307 Dresden, Germany

**Keywords:** Retroperitoneal lymph node dissection, Complications, Testicular cancer, Additional surgical procedures

## Abstract

**Purpose:**

Retroperitoneal lymph node dissection (RPLND) for germ cell tumours is a challenging procedure that may present relevant complications. The purpose was to analyse postoperative complications and identify risk factors for major complications.

**Methods:**

This is a retrospective unicentric analysis of a large cohort of 295 RPLNDs from 1992 to 2020. Early complications (30 days) and late complications (31–180 days) were classified according to the Clavien‒Dindo classification. The influence of surgical, patient-specific, and tumour-specific parameters on grade III–V complications was analysed in univariate and multivariate logistic regression models.

**Results:**

A total of 232 were postchemotherapy RPLNDs, and 63 were primary RPLNDs. Early postoperative complications were found to be grades I–II in 58.6% (173/295), grades III–IV in 9.8% (29/295), and grade V in 0.3% (1/295). In 20% (58/295), additional surgical procedures were needed. Grade III–V complications were associated with ≥ 4 cycles of preoperative chemotherapy (OR 3.7 (1.5–8.9); *p* = 0.004), RPLND specimen (nonseminoma or immature teratoma) (OR 3.1 (1.4–6.6); *p* = 0.005), transfusions (OR 2.4 (1.1–5.4); *p* = 0.03), salvage RPLND (OR 4.1 (1.8–9.3); *p* < 0.001), and preoperatively elevated AFP (OR 5 (2.2–11.7); *p* < 0.001). In multivariate analysis, the only independent predictor for grade III–V complications was preoperative AFP elevation (OR 3.3 (1.2–9.2); *p* = 0.02). Limitations include the retrospective study design.

**Conclusions:**

Our results demonstrate that RPLND is a demanding surgical procedure. Patients with a complex tumour history have a higher risk of complications. We recommend treatment of these complex cases in high-volume centres.

**Supplementary Information:**

The online version contains supplementary material available at 10.1007/s00345-023-04516-7.

## Introduction

Testicular germ cell tumours are the most common form of cancer in male patients in the age group between 20 and 40 years [1]. Even metastatic testicular germ cell tumours have high survival rates with low mortality [2]. However, advanced and recurrent tumours need complex therapy with cisplatin-based chemotherapy and retroperitoneal lymph node dissection (RPLND). Primary RPLND (pRPLND) is performed for diagnostic and therapeutic indications without prior chemotherapy [3, 4]. Due to few indications, pRPLND is rarely performed. Postchemotherapy RPLND (pcRPLND) is performed for resection of residual retroperitoneal masses after chemotherapy [4]. RPLND is a complex surgical procedure with a relevant complication rate [5–7]. In particular, pcRPLND is a technically challenging procedure because of tissue alterations resulting from prior chemotherapy [8–10]. Therefore, these dissections can lead to extended resection with removal of organs or vascular surgery [9]. The complication rate depends on surgical experience and hospital volume [11, 12]. Postoperative complications are indicated objectively with the Clavien‒Dindo classification [13]. There are several studies detailing major complications [6, 7, 9], but there is little information regarding minor complications (Clavien‒Dindo I & II), which would underline the demanding postoperative handling of RPLND patients and the need for more centralization. Furthermore, only a few studies consider late complications (> 30 days). A study analysing the German hospital billing database showed that 80.5% of all RPLND in Germany were performed in low- and medium-volume hospitals (≤ 9 cases/year) [12]. In high-volume hospitals (≥ 10 cases/year), patients had a shorter hospital stay despite more complex cases, reflecting the benefit of experience in the treatment of such cases. Early and late complications can influence the future life of these young patients. Thus, it is important to identify possible risk constellations, to guide more complex cases to high-volume centres, educate patients in a targeted manner, and address possible complications in a preventive manner. The aim of this retrospective study was to analyse the complications of RPLND in a large cohort and investigate parameters that may be associated with a higher risk of severe complications.

## Patients and methods

According to the EAU Guidelines Panel Assessment and Recommendation for Reporting and Grading of Complication, we report the following parameters [14]: We retrospectively analysed 307 RPLNDs performed for malignant germ cell tumours via chart review at the Department of Urology of the University Hospital Carl Gustav Carus in Dresden between 1992 and 2020. Patients who received only a pick-up RPLND for histological confirmation were excluded (*n* = 12). Data were collected by medical doctors involved in the treatment. The duration of complete follow-up was 30 days and included outpatient information. For duration of delayed complications (until 180 days), we had data to *n* = 221 patients. The definition of complications and procedure-specific complications as well as grading was defined according to the Clavien‒Dindo classification [13]. For every patient, all complications were coded. The most severe complication was defined by the Clavien‒Dindo classification. We defined prolonged catecholamine-usage (≥ 2 days after surgery) as Clavien‒Dindo grade 2. Findings of asymptomatic lymphoceles were defined as Clavien‒Dindo grade 1. Due to the homogenous collective (young male patients), risk factors were patient- and disease-specific parameters, such as age, body mass index (BMI), tumour stage, tumour-specific therapy, and laboratory parameters, including tumour markers (alpha-fetoprotein (AFP), human chorionic gonadotropin (ß-HCG), lactate dehydrogenase (LDH)). Furthermore, the duration of surgery as well as additional surgical procedures was determined. An additional procedure in addition to the regular RPLND was defined as a visceral resection (planned or unplanned) to remove residual tumour or to reconstruct vessels or upper urinary tract (orchiectomies were excluded). Reoperations and their causes were documented. There were no patients lost to follow-up 30 days after RPLND, but 74 patients lost to follow-up for late complication (> 30 days). Institutional review board approval was obtained for this study.

Data were analysed using the Chi-square test and t test. Binary logistic regression models were used for univariate and multivariate estimation of risks and to predict outcome events. We used the median of continuous variables (age, duration of surgery, removed lymph nodes) for grouping in binary logistic regression models; *p* < 0.05 was considered significant. All calculations were performed with IBM SPSS Statistics 28.0 (Armonk, New York, USA).

## Results

An analysis of 295 RPLNDs performed for germ cell tumours at our hospital between 1992 and 2020 was conducted. Sixty-three were primary RPLND (pRPLND), and 232 were postchemotherapy RPLND (pcRPLND), with 5 patients having received a prior RPLND and thus having a second or third procedure. Forty-four pcRPLNDs were salvage RPLNDs. The mean age was 33 years (IQR 16–70). A total of 94% (261/278) had nonseminoma, and 6% (17/278) had seminoma. Early postoperative complications (within 30 days) according to the Clavien‒Dindo classification were found to be grades I–II in 58.6% (173/295), III–IV in 9.8% (29/295), and V in 0.3% (1/295). There were more complications in pcRPLND (92% vs. 62%; *p* > 0.001) (Table 1).

**Table 1:** Patient characteristics by RPLND type (*n* = 295)

Variables	All (*n* = 295)	pcRPLND (*n* = 232)	pRPLND (*n* = 63)	*p* value
Age (years) [mean SD, median (IQR)]				
	33.1 ± 10.2	33.4 ± 10.3	31.8 ± 9.7	0.3
	32 (16–70)	32 (16–70)	31 (16–63)	
BMI (kg/m^2^) [mean SD, median (IQR)]				
	24.8 ± 4.3	24.8 ± 4.6	24.6 ± 2.9	0.6
	24.4 (16.6–49.5)	24.3 (16.6–49.5)	24.9 (18.0–32.5)	
Primary histology (*n* = 278)				
Seminoma	17 (6%)	15 (7%)	2 (3%)	0.2
Nonseminoma	261 (94%)	201 (93%)	60 (97%)	
Preoperative AFP (*n* = 292) [mean SD, median (IQR)]				
	83.9 ± 961.7	104.4 ± 1080.8	6.0 ± 13.9	0.5
	3.1 (0.2–15,776.0)	3.3 (0.7–15,776.0)	2.8 (0.2–106.6)	
Preoperative HCG (*n* = 291) [mean SD, median (IQR)]				
	14.1 ± 143.1	17.5 ± 160.8	1.4 ± 4.8	0.4
	0 (0–2090.0)	0 (0–2090.0)	0 (0–25.6)	
Preoperative LDH (*n* = 264) [mean SD, median (IQR)]				
	4.3 ± 1.4	4.3 ± 1.5	4.4 ± 1.2	0.5
	4.0 (2.2–9.8)	3.8 (2.2–9.8)	4.5 (2.7–7.9)	
RPLND side (*n* = 290)				
Right	11 (4%)	6 (3%)	5 (8%)	0.01
Left	20 (7%)	12 (5%)	8 (13%)	
Bilateral	259 (89%)	211 (92%)	48 (79%)	
Operating time (n = 294) [mean SD, median (IQR)]				
	325.1 ± 117.7	342.5 ± 119.4	259.9 ± 84.2	< 0.001
	306 (38–790)	319 (38–790)	238 (130–520)	
Blood transfusion (*n* = 295) [mean SD, median (IQR)]				
	2.2 ± 3.8	2.8 ± 4.1	0.1 ± 0.4	< 0.001
	0 (0–23)	2 (0–23)	0 (0–2)	
Additional surgical procedure				
No	237 (80%)	187 (81%)	50 (79%)	0.9
Yes	58 (20%)	45 (19%)	13 (21%)	
Number of removed lymph nodes (*n* = 249) [mean SD, median (IQR)]				
	25.1 ± 13.1	25.6 ± 13.2	23.4 ± 12.7	0.3
	23 (1–70)	24 (1–60)	20 (7–70)	
Positive lymph nodes (*n* = 289) [mean SD, median (IQR)]				
	1.3 ± 3.3	1.6 ± 3.7	0.4 ± 0.7	< 0.001
	0 (0–29)	0 (0–29)	0 (0–2)	
RPLND Specimen				
No tumour	157 (53%)	114 (49%)	43 (68%)	< 0.001
Mature teratoma	65 (22%)	64 (28%)	1 (2%)	
Immature teratoma and nonseminoma	73 (25%)	54 (23%)	19 (30%)	
Clavien- Dindo classification				
0	122 (41%)	69 (30%)	53 (84%)	< 0.001
1–2	143 (48%)	136 (58%)	7 (11%)	
3–4	29 (10%)	26 (11%)	3 (5%)	
5	1 (1%)	1 (1%)	0 (0%)	
Preoperative number of chemotherapy cycles				
0	63 (21%)	0 (0%)	63 (100%)	< 0.001
1–3	84 (29%)	84 (36%)	0 (0%)	
≥ 4	148 (50%)	148 (64%)	0 (0%)	
Salvage RPLND				
No	250 (85%)	187 (81%)	63 (100%)	< 0.001
Yes	44 (15%)	44 (19%)	0 (0%)	
Prognosis group (*n* = 294)				
Good	175 (60%)	115 (50%)	60 (95%)	< 0.001
Intermediate	60 (20%)	57 (25%)	3 (5%)	
Poor	59 (20%)	59 (25%)	0 (0%)	

The most common major complication was lymphoceles, which had to be treated as an intervention. There was one death because of acute leg ischaemia with a complicated course. The most common minor complications were postoperative transfusions (*n* = 117) and prolonged catecholamine usage (*n* = 97) (Supplementary Tables 1 & 2). In 58/295 (20%) cases, resections of further organs or vascular reconstructions were needed. The most common procedures were vascular reconstruction (17/295), nephrectomy (10/295), and adrenalectomy (10/295) (Supplementary Table 3).

In univariate analysis, patients with grade III–V complications showed a longer operating time (371.8 ± 124.9 vs. 319.8 ± 115.9 min.; *p* < 0.02), more blood transfusions (4.8 ± 5.6 vs. 1.9 ± 3.4; *p* < 0.001), a high number of prior chemotherapy cycles (≥ 4 cycles: 77% vs. 47%; *p* = 0.002), more frequent salvage RPLND (37% vs. 12%; *p* < 0.001), AFP elevation preoperatively (37% vs. 10%, *p* < 0.001), and detection of nonseminoma or immature teratoma in the RPLND specimen (43% vs. 20%; *p* = 0.003) (Table 2).

**Table 2:** Patient and surgical characteristics by Clavien‒Dindo classification in 295 RPLNDs

Variables	Clavien‒Dindo 0 – II (*n* = 265)	Clavien‒Dindo III – V (*n* = 30)	*p* value
Age (years) [mean SD, median (IQR)]			
	32.7 ± 9.6	36.3 ± 14.5	0.2
	32 (16–66)	34 (16–70)	
BMI (kg/m^2^) [mean SD, median (IQR)]			
	24.7 ± 4.3	25.0 ± 4.1	0.7
	24.4 (16.6–49.5)	24.7 (19.1–35.9)	
Primary histology (*n* = 278)			
Seminoma	15 (6%)	2 (7%)	0.9
Nonseminoma	235 (94%)	26 (93%)	
Operating time (*n* = 294) [mean SD, median (IQR)]			
	319.8 ± 115.9	371.8 ± 124.9	0.02
	300 (38–790)	348 (192–630)	
Blood transfusion (*n* = 295) [mean SD, median (IQR)]			
	1.9 ± 3.4	4.8 ± 5.6	
	0 (0–23)	2 (0–18)	< 0.001
Additional surgical procedure			
No	225 (85%)	24 (80%)	0.5
Yes	40 (15%)	6 (20%)	
Number of removed lymph nodes (*n* = 249) [mean SD, median (IQR)]			
	25.0 ± 13.0	26.0 ± 14.2	0.7
	23 (1–60)	24 (1–70)	
RPLND Specimen			
No tumour or mature teratoma	212 (80%)	17 (57%)	0.004
Immature teratoma and nonseminoma	53 (20%)	13 (43%)	
Preoperative number of chemotherapy cycles			
0–3	140 (53%)	7 (23%)	0.002
≥ 4	125 (47%)	23 (77%)	
Salvage RPLND			
No	232 (88%)	19 (63%)	< 0.001
Yes	33 (12%)	11 (37%)	
Preoperative elevated AFP (*n* = 292)			
Yes	27 (10%)	11 (37%)	< 0.001
No	235 (90%)	19 (63%)	
Preoperative elevated HCG (*n* = 291)			
Yes	22 (8%)	4 (14%)	0.3
No	241 (92%)	24 (86%)	
Preoperative elevated LDH (*n* = 264)			
Yes	131 (56%)	13 (43%)	0.2
No	103 (44%)	17 (57%)	

In binary logistic regression, the following parameters were significant in univariate analysis for grade III–V complications: high blood transfusion (OR 2.4 (1.1–5.4); *p* = 0.03), ≥ 4 cycles of prior chemotherapy (OR 3.7 (1.5–8.9); *p* = 0.004), nonseminoma or immature teratoma in the RPLND specimen (OR 3.1 (1.4–6.6); *p* = 0.005), salvage RPLND (OR 4.1 (1.8–9.3); *p* < 0.001), and AFP elevation preoperatively (OR 5 (2.2–11.7); *p* < 0.001). In multivariate analysis, the only independent predictor for grade III–V complications was AFP elevation preoperatively (OR 3.3 (1.2–9.2); *p* = 0.02) (Table 3).

**Table 3:** Univariate and multivariate binary regression analysis of risk factors for grade III–V complications in 295 RPLNDs

	Univariate		Multivariate	
	OR (95% CI)	*p* value	OR (95% CI)	*p* value
Preoperative chemotherapy (≥ 4 cycles)	3.7 (1.5–8.9)	0.004	2.0 (0.7–5.7)	0.1
RPLND specimen (Nonseminoma & immature Teratoma)	3.1 (1.4–6.6)	0.005	1.9 (0.8–4.3)	0.1
Additional surgical procedure	1.4 (0.5–3.7)	0.5		
Age (≥ 31 years)	1.6 (0.7–3.5)	0.3		
BMI (≥ 30)	0.9 (0.2–3.0)	0.8		
Operation time (≥ 306 min)	2.2 (1.0–4.8)	0.06		
Transfusion	2.4 (1.1–5.4)	0.03	1.5 (0.6–3.7)	0.4
Removed lymph nodes (≥ 23)	1.5 (0.5–2.3)	0.9		
Tumour in specimen	2.2 (1.0–4.9)	0.054		
RPLND type (pcRPLND)	1.6 (0.9–3.0)	0.1		
Salvage RPLND	4.1 (1.8–9.3)	< 0.001	1.4 (0.5–4.2)	0.6
Preoperatively elevated AFP	5 (2.2–11.7)	< 0.001	3.3 (1.2–9.2)	0.02
Preoperatively elevated HCG	1.8 (0.6–5.7)	0.3		
Preoperatively elevated LDH	0.6 (0.3–1.3)	0.2		

Periodical analysis of early complications shows a difference over time. Between 1992 and 2002, there were 4.4% (6/137) grade III–V complications. Since 2003, there were 15.2% (24/158) grade III–V complications. Focusing on pcRPLND, between 1992 and 2002 there were 5.6% grade III–V complications, and since 2003, there were 15.4% grade III–V complications (Supplementary Figs. 1 & 2).

Regarding delayed (until 180 d) complications, we had data of 221 RPLNDs. After 30 days, 33 patients showed grade I–II complications and 15 patients grade III complications (Supplementary Tables 4 & 5).

## Discussion

This study shows a complication rate of 48.5% grade I–II and 10.1% grade III–V for early complications (within 30 days) in a high-volume hospital. In 20% of cases, additional procedures were needed. In multivariate analysis, preoperatively increased AFP was an independent parameter for grade III–V complications (OR 3.8 (1.6–9.4); *p* = 0.003).

Evaluating grade I–II complications and discussing them with other studies are difficult. One reason may be incomplete documentation of grade I–II complications in daily clinical routine. Another reason is the imprecise wording of Clavien‒Dindo grade I. There it is stated “Any deviation from the normal postoperative course…” [13]. However, there is no definition of deviations regarding the normal postoperative course of RPLND. There are only a few studies analysing minor complications. Our study showed 48.5% (143/295) Clavien‒Dindo grade I–II complications. Grade II complications were mainly prolonged catecholamine usage (33%; 97/295) and transfusions (40%; 117/295). Grade I complications were mostly asymptomatic lymphoceles (16%; 48/295). In comparison, other studies show fewer minor complications. A German study showed only 6% surgery-associated Clavien‒Dindo grade II and no grade I complications [9]. However, it is not specified what kind of complications they defined as grade II. Additionally, a large US study showed only 2% grade I and II complications, detailing kinds of complications but without mentioning transfusions [6]. In contrast, a Scandinavian study showed 34% grade I–II complications, which corresponds with our study [15]. Finally, it is difficult to interpret grade I–II complications because of the limited number of studies with inhomogeneous documentation. Furthermore, it is discussed whether only the most severe or all complications should be documented [16]. We think it is important to analyse grade I–II complications to point out that RPLND is also challenging in postoperative handling.

Several studies have analysed the early complication rate in RPLND, with grade III–IV complications varying between 1.3 and 23.3% [6, 17]. Thus, our complication rate of 9.8% grade III–IV fits the complication rates of high-volume centres. Nevertheless, centres with a very high volume in the USA with more centralization of RPLND show better complication rates of 1.2–1.3% [6, 11, 18]. This underlines the advantage of centralization with high-volume centres. In our cohort, there was one death resulting in 0.3% grade V complications. This is congruent with an international study, where grade V complications ranged between 0 and 1% [7]. All of these studies analysed open RPLND. In recent years, we have seen a small but continuously increasing number of minimally invasive RPLNDs driven by the robotic approach [12]. There are several studies concerning complications in robotic RPLND, but a comparison to the established open procedure is difficult because of a selective collective of less complex cases [19, 20].

The most common grade III–IV complication in our study was symptomatic lymphoceles (17%, *n* = 5/30). In this study, 17.9% (53/295) of patients had findings of lymphoceles or lymph ascites. These findings range between a few centimetres for small lymphoceles and large symptomatic lymph ascites where an intervention was needed. Therefore, it is difficult to compare these asymptomatic findings with other studies where lymphocele formation ranged between 0 and 14.6% [21, 22].

The rate of additional surgical procedures is another parameter describing the surgical complexity of RPLND in addition to the complication rate. In 20% (58/295), additional surgical procedures were needed. This corresponds to other studies where additional procedures range between 13 and 22% [6, 9, 15].

Parameters associated with major complications can be classified into three groups. The first is parameters reflecting a complex surgical procedure (transfusion rate, duration of surgery). The second is parameters reflecting extension of surgery (side of RPLND, additional procedures). The third is patient-related parameters (age, BMI, tumour situation). Regarding the first group, a long duration of surgery (*p* = 0.02) and a high transfusion rate (*p* < 0.001) were associated with major complications. Another recent German study showed the same finding regarding the duration of surgery [7]. Interestingly, the extent of surgery in the form of additional procedures or extension of the resection area was not associated with major complications. Additionally, two German studies could not find an association of major complications with the extension of lymph node dissection [7, 23]. In contrast, a Scandinavian study showed more major complications in bilateral resection than in unilateral resection [15]. Studies analysing the surgical management of complex residual masses comparing RPLND without additional resections show more major complications in the resection of complex residual masses [6, 9]. These studies focused on complex residual masses, which seemed to be more extensive than in our collective. The third group of parameters was patient related. Here, common factors such as age and BMI were not associated with major complications, which corresponds with another German study with 146 pcRPLNDs [7]. In contrast, cancer-specific factors associated with major complications were aggressive histology in RPLND specimens (nonseminoma and immature teratoma *p* = 0.004), more than four cycles of chemotherapy (*p* = 0.002), salvage RPLND (*p* < 0.001), and a preoperatively elevated AFP (*p* < 0.001). Interestingly, in binary multivariate analysis of the already mentioned parameters, only preoperatively elevated AFP was an independent parameter associated with major complications. Connections between tumour- and therapy-specific parameters were also found in the past, but the results were ambiguous. A Canadian study showed more complications in patients with fibrosis and large tumours [24]. An US study showed more complications in patients with primary pure seminoma than in patients with seminomatous elements [8]. The German study showed more complications in patients with viable cancer in contrast to teratoma or necrosis/fibrosis [7]. A US study showed elevated tumour markers and vital cancer in specimens as independent predictors for additional procedures during pcRPLND [6]. This is the only study including tumour markers in the analysis of surgical outcomes. Nevertheless, there are no comparable studies regarding the association of major complications with tumour-specific parameters. Thus, it remains speculative why AFP is an independent predictor of major complications in this study. We assume that patients with preoperatively increased AFP have more tissue alterations resulting from previously received chemotherapy and vital nonseminomatous tumour tissue.

Regarding late complications, 15/221 (6.8%) patients had grade III complications. There is little information in the literature regarding late complications. A study from Indianapolis showed 7% of late complications in both pRPLND and pcRPLND, which corresponds to our data [21]. The German Testicular Study Cancer Group reported an overall late complication rate with 4.4% [25].

Over the study period of almost 30 years, we can see changes in RPLND. There is a decrease in yearly RPLNDs. A study analysing numbers of RPLNDs in Germany between 2006 and 2015 shows a decrease of 61% [12]. This reflects the introduction of FDG-PET in diagnostic of seminoma tumour mass and changes in guidelines regarding the interpretation of residual mass in nonseminoma [26, 27]. The increase in major complications (4.4% between 1992 and 2002; 15.2% between 2003 and 2020) may result because of the reduction in indication in guidelines reducing RPLNDs to more complex cases. Nevertheless, the almost vanishing of pRPLNDs had no influence (pcRPLND: 5.6% (1992–2002) vs. 15.4% (2003–2020)). The decrease in yearly number of RPLNDs may also influence routine and experience.

The limitation of this study is the retrospective analysis of a unicentric cohort. In retrospective analysis, there is concern for incomplete documentation resulting in difficulties noticing complications. For this study, we analysed patient files. Due to quality management and billing in Germany, it is very unlikely that there are missing data regarding pharmacological and surgical interventions in patient files. Only grade I complications have a higher risk of missing data because they do not lead to interventions that require documentation. Nevertheless, few studies have focused in detail on complications after RPLND. Our study shows complications after RPLND in a large cohort of 295 cases in a very detailed way. This shows that the surgical procedure (20% multivisceral resections or vascular reconstruction) and postoperative course (48% grade I–II complications) are demanding. Nevertheless, it also shows a low complication rate (10.1% grade III–V) in a high-volume hospital with experience and infrastructure to perform this demanding surgical procedure. In particular, complex tumour cases are predestined for this. The study underlines the importance for centralization to perform RPLND in high-volume centres—at least for complex tumour cases.

## Conclusions

RPLND is a complex surgical procedure that often requires additional surgical procedures. In particular, patients after chemotherapy with vital residual tumours have more grade III–V complications, whereas a preoperatively increased AFP is an independent parameter. RPLNDs should be performed in high-volume centres with experience to perform additional multivisceral resections and a low complication rate.

## Supplementary Information

Below is the link to the electronic supplementary material.

Supplementary file1 (DOCX 21 KB)

Supplementary file2 (DOCX 17 KB)

## Data Availability

The data that support the findings of this study are available from the corresponding author upon reasonable request.
