# Supplementary material for: Retroperitoneal lymph node dissection for testicular cancer is a demanding procedure: detailed real-life data of complications and additional surgical procedures in 295 cases
Source: World J Urol. 2023 Jul 25;41(9):2397–404. doi: 10.1007/s00345-023-04516-7 (PMC10465663; doi:10.1007/s00345-023-04516-7)
Supplement: Supplementary file 1 — Supplementary file1 (DOCX 21 KB) [file 345_2023_4516_MOESM1_ESM.docx]

**Supplementary Figures**

Suppl. Figure 1: Clavien Dindo Grading according the year of surgery.

Suppl. Figure 2: Type and number of RPLND according the year.

Suppl. Figure 1: Clavien Dindo Grading according the year of surgery.

Suppl. Figure 2: Type and number of RPLND according the year.
