# Supplementary material for: Retroperitoneal lymph node dissection for testicular cancer is a demanding procedure: detailed real-life data of complications and additional surgical procedures in 295 cases
Source: World J Urol. 2023 Jul 25;41(9):2397–404. doi: 10.1007/s00345-023-04516-7 (PMC10465663; doi:10.1007/s00345-023-04516-7)
Supplement: Supplementary file 2 — Supplementary file2 (DOCX 17 KB) [file 345_2023_4516_MOESM2_ESM.docx]

**Supplementary Tables**

Suppl. Table 1: All complications according to the Clavien‒Dindo classification in 295 RPLNDs.

Suppl. Table 2: Reoperations because of complications in 295 RPLNDs.

Suppl. Table 3: Additional surgical procedures during RPLND in 46 patients.

Suppl. Table 4: Delayed complications (day 31 – 180) according to the Clavien‒Dindo classification in n=221 RPLNDs.

Suppl. Table 5: Reoperations because of delayed complications (day 31 – 180) in n=221 RPLNDs.

| **Grade I – II complications** | | **Grade III – V complications** | |
| --- | --- | --- | --- |
| Postoperative blood transfusions | 117 (68%) | Symptomatic lymphoceles | 5 (17%) |
| Catecholamine demand (≥2 days) | 97 (56%) | Wound dehiscence | 5 (17%) |
| Asymptomatic lymphoceles | 12 (7%) | Upper gastrointestinal bleeding | 4 (13%) |
| Subileus | 12 (7%) | Ectasia of renal pelvis | 4 (13%) |
| Infection (pulmonary 4; intestinal 1; unknown 6) | 11 (6%) | Haemorrhage | 3 (10%) |
| Wound healing problems | 6 (3%) | Acute pancreatitis | 2 (7%) |
| Haematoma | 4 (2%) | Pneumothorax | 2 (7%) |
| Decubitus | 4 (2%) | Ileus | 1 (3%) |
| Temporary neurological deficits | 4 (2%) | Dislocated port catheter | 1 (3%) |
| Transition syndrome | 2 (1%) | Acute appendicitis | 1 (3%) |
| Urinary retention | 2 (1%) | Intraabdominal abscess | 1 (3%) |
| Cardiac arrhythmia | 2 (1%) | Leg ischaemia with compartment syndrome | 1 (3%) |
| Leg lymph oedema | 2 (1%) | Acute renal dysfunction | 1 (3%) |
| Leg thrombosis | 2 (1%) | Prolonged intubation | 1 (3%) |
| Mild myocardial infarction | 1 (1%) | Ischaemia right kidney | 1 (3%) |
| Pleural effusion | 1 (1%) |  |  |
| Pulmonary embolism | 1 (1%) |  |  |
| Unknown decrease of oxygen saturation | 1 (1%) |  |  |
| Leukopenia | 1 (1%) |  |  |
| Temporary creatinine elevation | 1 (1%) |  |  |

Suppl. Table 1: All complications according to the Clavien‒Dindo classification in 295 RPLNDs.

| **Complication** | **Reoperation** | |
| --- | --- | --- |
| Symptomatic lymphoceles | Lymphocele drainage | 5 |
| Wound dehiscence | Secondary wound closure | 5 |
| Upper gastrointestinal bleeding | Gastroscopy with clipping | 4 |
| Ectasia of renal pelvis | Ureteral stenting | 4 |
| Haemorrhage | Laparotomy with haemostasis | 3 |
| Pneumothorax | Pleural drainage | 2 |
| Ileus | Laparotomy | 1 |
| Dislocated port catheter | Port removal | 1 |
| Acute appendicitis | Appendectomy | 1 |
| Intraabdominal abscess | Laparotomy with abscess removal | 1 |
| Leg ischaemia with compartment syndrome | Embolectomy with compartment spading of the leg | 1 |

Suppl. Table 2: Reoperations because of complications in 295 RPLNDs.

| **Additional surgical procedures (56 procedures in 46 patients)** | |
| --- | --- |
| Vascular reconstruction | 17 (37%) |
| Nephrectomy | 10 (22%) |
| Adrenalectomy | 10 (22%) |
| Liver resections | 6 (13%) |
| Bowel resections | 5 (11%) |
| Reconstructions ureter/renal pelvis | 3 (7%) |
| Splenectomy | 2 (4%) |
| Pancreas tail resection | 1 (2%) |
| Urachal cyst resection | 1 (2%) |
| Radical prostatectomy | 1 (2%) |

Suppl. Table 3: Additional surgical procedures during RPLND in 46 patients.

| **Grade I – II complications (n=33)** | | **Grade III – V complications (n=15)** | |
| --- | --- | --- | --- |
| Scar pain | 16 (48%) | Ectasia of renal pelvis | 7 (64%) |
| Asymptomatic lymphoceles | 12 (36%) | Symptomatic Lymphocele | 3 (27%) |
| Leg thrombosis | 4 (12%) | Wound dehiscence | 2 (18%) |
|  |  | Loss of kidney function | 1 (10%) |
|  |  | Symptomatic Lymphocele | 1 (10%) |
|  |  | Abscess | 1 (10%) |

Suppl. Table 4: Delayed complications (day 31 – 180) according to the Clavien‒Dindo classification in n=221 RPLNDs.

| **Complication** | **Reoperation** | |
| --- | --- | --- |
| Ectasia of renal pelvis | Ureteral stenting | 7 |
| Wound dehiscence | Secondary wound closure | 2 |
| Symptomatic Lymphocele | Laparotomy with resection | 2 |
| Symptomatic Lymphocele | Lymphocele drainage | 1 |
| Abscess | Abscess drainage | 1 |
| Symptomatic Lymphocele | Lymphocele drainage | 1 |
| Loss of kidney function | nephrectomy | 1 |

Suppl. Table 5: Reoperations because of delayed complications (day 31 – 180) in n=221 RPLNDs.
